# PReS-FINAL-2271: Assessment of musculoskeletal abnormalities in children with mucopolysaccharidoses using a simple musculoskeletal examination (paediatric gait, arms, legs and spine)

**DOI:** 10.1186/1546-0096-11-S2-P261

**Published:** 2013-12-05

**Authors:** MO Chan, ES Sen, E Hardy, T Rapley, P Hensman, E Wraith, H Foster

**Affiliations:** 1 Paediatric Rheumatology, University of British Columbia, Vancouver, Canada; 2 Paediatric Rheumatology, Great North Children's Hospital, Newcastle upon Tyne, UK; 3 Institute of Health and Society, Newcastle University, Newcastle upon Tyne, UK; 4 Manchester Children's Hospital, Manchester, UK; 5 Musculoskeletal Research Group, Institute of Cellular Medicine, Newcastle University, Newcastle upon Tyne, UK

## Introduction

Mucopolysaccharidoses (MPS) are rare inherited disorders with a spectrum of phenotypes resulting from glycosaminoglycans (GAGs) accumulation in cells. Children with MPS often have musculoskeletal (MSK) abnormalities ranging from joint contractures to deforming abnormalities of the extremities and spine. pGALS (paediatric Gait, Arms, Legs, and Spine), is a simple MSK assessment which has been previously validated in school-age children to detect abnormal joints.

## Objectives

Our aim was to describe the use of pGALS to identify patterns of MSK abnormalities in children with MPS.

## Methods

Videos of children with a spectrum of MPS performing pGALS were made at an MPS specialist centre as part of their routine care and independent of the current study. Informed consent for the use of the videos for research was obtained. A piloted proforma to record abnormalities for each pGALS manoeuvre observed in the videos (scored as normal/abnormal/not assessable) was used by 3 observers (2 paediatric rheumatology (PRh) trainees and 1 specialist PRh physiotherapist and all blinded to MPS subtype). Videos were scored independently by the 3 observers and videos re-scored for intra- and inter-observer consistency. Data were pooled and analysed.

## Results

15 videos of children (9 boys, 6 girls, median age 11 years [4-19]) with MPS (10 MPS type I Hurler-Scheie (HS); 4 MPS type II; 1 mannosidosis) were assessed. The most common abnormalities detected using pGALS exam were restriction of shoulder, elbow, wrist, temporomandibular joint excursion (likely impacted by many children having enlarged digits) [>75% cases] and spinal deformity/restriction (2/3 cases). Mean intra-observer Kappa 0.74 (range 0.65-0.88) and inter-observer Kappa 0.62 (range 0.51-0.77). Hip manoeuvres within pGALS were not clearly demonstrated in the videos.

## Conclusion

In this observational study, pGALS identifies MSK abnormalities in children with MPS. Restricted joint movement (and especially upper limb) was a consistent finding. We acknowledge that further work is needed to include pGALS assessment of the hip and also to test pGALS in an additional population of children with MPS; notably in further children with MPS I-HS as this subtype often has MSK abnormalities as the only feature. The use of pGALS and awareness of patterns of joint involvement may be a useful adjunct to facilitate earlier recognition of these rare conditions and facilitate access to specialist care.

## Disclosure of interest

None declared.

